# Endocarditis

**Published:** 1897-06-19

**Authors:** 


### ENDOCARDITIS.

In a lecture on endocarditis recently delivered by Dr. Lauder Brunton at St. Bartholomew's Hospital, there were three little "tips" which may usefully be remembered.

The first is the importance of keeping a careful watch on the temperature in this disease. In the diagnosis of endocarditis the thermometer is of as much use, or even of more use than the stethoscope. But it is not enough to take the temperature at long intervals, and especially it is not enough to take it at one fixed time every day. What happens in endocarditis is that the temperature runs up occasionally for short periods, so that the curve may be very apparent in a four-hour chart, but may not show at all on an ordinary daily chart.

Endocarditis may occur from various causes, and one form, the microbial or so-called ulcerative endocarditis, is not unfrequently mistaken for typhoid fever. Hence the importance of making frequent observations of the temperature in a doubtful case of typhoid, lest perchance it might happen to be one of endocarditis. A four hours' chart may give a clue to the diagnosis in a case which might remain quite obscure if the temperature were taken only night and morning. Dr. Brunton says, "If you find a murmur at the heart, or even in cases where you find no murmur at the heart, if you find a temperature which runs a course very much like that of quotidian ague, in a case where you can trace no malaria, and where you can find no indication of suppuration, it is very likely indeed to be a case of endocarditis."

The next point is a recommendation as to the use of benzoate of soda in cases of malignant endocarditis, made to Dr. Brunton by the late Sir Andrew Clarke; and lastly comes the suggestion that cases of endocarditis are peculiarly apt to occur at "the fall of the leaf," coupled with an expression of a strong belief that it is advisable for delicate people to avoid fallen leaves as much as they can.

